# Resilience of seafarers depending on occupational groups

**DOI:** 10.1186/s12889-026-27133-6

**Published:** 2026-03-31

**Authors:** Wiebke Janssen, Hans-Joachim Jensen, Volker Harth, Marcus Oldenburg

**Affiliations:** Institute for Occupational and Maritime Medicine Hamburg (ZfAM), University Medical Center Hamburg-Eppendorf (UKE), Hamburg, 20459 Germany

**Keywords:** Seafaring, Resilience, Stress, Depression, Occupational groups

## Abstract

**Background:**

Seafaring is a high-stress occupation that can significantly impact the mental health of crew members. Understanding the associations between resilience, stress perception, and depression is crucial for developing effective interventions to support their well-being. This study aims to investigate these relationships and explore potential differences between officers and ratings in resilience, stress perception, and depression.

**Methods:**

A total of 902 participants, comprising officers and ratings, provided information on their resilience, stress perception, and depression using standardized instruments. Descriptive statistics were employed to analyze the data, including Chi-squared tests and logistic regression to assess differences between occupational groups.

**Results:**

Officers demonstrated higher resilience significantly more often compared to ratings (OR 1.74; 95% CI (1.27–2.39)). Conversely, ratings more often reported moderate to high levels of stress perception compared to officers (64.2% vs. 53.3%; *p* = 0.001). However, no significant difference was observed in depression rates between the two groups. Various factors such as age, duration on board, family status, and cultural background influence the resilience, stress perception, and depression rates of seafarers. Seafarers from Europe (78.6% vs. 70.3%; *p* = 0.023) and those with easy access to music on board (75.7% vs. 39.3%; *p* < 0.001) tend to endorse higher resilience more often. These cross-sectional findings provide an observation of associations between resilience, stress perception, and depression, recognizing that resilience is a dynamic process that evolves over time.

Higher resilience was associated with lower levels of depressive symptoms (Spearmans ρ=-0.199, *p* < 0.001) and lower perceived stress (Spearmans ρ=-0.305, *p* < 0.001). In contrast, higher stress perception was strongly associated with greater depressive symptoms (Spearmans ρ = 0.333, *p* < 0.001).

**Conclusions:**

The study highlights the interrelatedness of resilience, stress, and mental health among seafarers, suggesting that resilience may help buffer the impact of stress. The findings suggest that supporting the ongoing development of resilience and managing stress are important for promoting mental well-being among seafarers. Further research is warranted to explore the longitudinal nature of resilience additional factors influencing resilience among seafarers and develop evidence-based interventions to support their mental health needs.

**Supplementary Information:**

The online version contains supplementary material available at 10.1186/s12889-026-27133-6.

## Background

In todays world, mental health is gaining increasing importance as it significantly influences our well-being. Resilience is a key factor in mental health, as it determines how well someone copes with crises and the extent to which they leave sustainable damages, especially in terms of mental health. Resilience, as described by the Leibniz Institute for Resilience (LIR) Research in Germany, refers to the capacity of individuals and communities to confront and adapt to adversity, involving extracting valuable lessons from challenges and setbacks [1]. It involves the ability to recover from stress and to sustain or regain both well-being and functionality. This definition underscores resilience as a complex and evolving process, encompassing not only the ability to cope with challenges and stressors, but also emphasizing learning and personal growth. This is particularly relevant in professions where people are frequently confronted with crises and challenging conditions.

In shipping, the significance of resilience holds a prominent position due to the unpredictable nature of the maritime environment, isolation at sea, and dependence on complex technology inherently making seafaring risky [2–4]. Officers and crew members may encounter various challenges that impact their mental health and resilience differently. It is essential to consider the possible positive influence of resilience on seafarers, as their demanding and often hazardous work, including stressful events, can expose them to high levels of stress. When people are exposed to prolonged periods of stress, they face a significant risk of developing depression [5, 6]. A resilient mindset, according to studies such as Ju-Yu Yen et al. (2019), could mitigate the potential onset of depression triggered by elevated stress levels [7]. 

Resilience in seafaring populations encompasses the ability of seafarers to adapt to adverse circumstances, recover, and operate successfully and therefore possibly reduce the probability of experiencing depression triggered by stress [7]. Considering the multicultural constellation of numerous ship crews, it is probable that cultural variations in resilience, stress perception, and depression exist. This hypothesis suggests that these differences may be present on board, influenced by factors like years of experience, age, and other demographic variables.

Job related differences between occupational groups play a vital role in understanding how resilience, stress and depression manifest within the maritime sector. Officers engaged with complex decisions and responsibilities may face distinct stressors compared to crew members, who are frequently exposed to physical exertion and challenging working conditions [8]. Additionally, the differing durations of time spent on board by officers and crew members may also impact their susceptibility to stress and depression.

When facing natural disasters, equipment failures, operational disruptions, or even piracy, resilience could be the key not only to overcome such challenges, but also to minimize their lasting impact. With the ongoing development of the maritime industry, the integration of modern technologies and the tackling of new environmental and geopolitical challenges, the importance of resilience in the maritime sector is becoming increasingly evident. This paper presents, as the first evaluation approach worldwide, a pilot study with a cross-sectional design aiming to explore the significance of resilience among seafarers and the correlation between resilience, stress, and depression in shipping crews, taking into account differences between occupational groups and other factors within the maritime sector. Understanding these associations is crucial for developing targeted interventions and support systems to enhance the mental well-being and resilience of shipping crews.

## Methods

In a previous systematic review published in 2024 the authors analyzed the relevance about resilience in maritime context [9]. For the present study a cross-sectional design was chosen to gain an overview of resilience among seafarers and to evaluate differences between occupational groups. Data collection was conducted using an electronic questionnaire that could be filled out by seafarers themselves. The survey was distributed to 1,299 crew members on 65 German merchant vessels. The study received ethical approval from the ethics committee of the Hamburg Medical Association (PV7174-4572-BO-ff).

### Study population

The questionnaire was completed by a total of 994 seafarers from one German shipping company, resulting in a response rate of 76.5%. 929 seafarers provided information about their rank and were therefore included for analysis in this study. An overview of the demographic characteristics of the study population, including age, gender, family status, and time on board, is provided in Table 1. Among the surveyed seafarers, there were only five women (0.5%), and the remaining 923 were men, one person did not give information about the gender. Age was originally recorded as an open-ended variable but was categorized into a total of six groups (19 or younger; 20–29 years; 30–39 years; 40–49 years; 50–59 years and 60–69 years) to enable group comparisons. The most prominent age group was 30–39 years (33.3%). For greater statistical power and clarity in reporting, the age distribution was indicated here in binary, with the lower three age groups and the upper three age groups being combined (< and ≥ 40 years). Regarding nationality, the classification into “European” and “Non-European” was made to reflect cultural and socioeconomic differences that may influence coping styles and access to mental health resources. This distinction is common in research on multicultural populations and has been used in previous studies as well [8]. 28.3% of the total sample were Europeans. The most represented nationality was Filipino at 53.4%, followed by Ukraine at 6.7%. In the officers’ group, the proportion of Europeans was 65.1%, while in the ratings’ group it was 4.4%.

**Table 1:** Demographic data of the study sample

	Total sample(n = 929)	Ratings(n = 567)	Officers (n = 362)
Gender, n (%)			
Male	923 (99.5%)	564 (99.5%)	359 (99.4%)
Female	5 (0.5%)	3 (0.5%)	2 (0.6%)
Age, n (%)			
< 40 years	498 (55.1%)	284 (51.8%)	214 (60.3%)
≥ 40 years	405 (44.9%)	264 (48.2%)	141 (39.7%)
Origin, n (%)			
European	210 (28.3%)	20 (4.4%)	190 (65.1%)
Non-European	532 (71.7%)	430 (95.6%)	102 (34.9%)
Family status, n (%)			
Single	207 (22.8%)	134 (24.2%)	73 (20.7%)
Married/Partnership	684 (75.5%)	411 (74.2%)	273 (77.6%)
Divorced/Separated	15 (1.7%)	9 (1.6%)	6 (1.7%)
Children, n (%)			
No	284 (31.2%)	161 (29.0%)	123 (34.6%)
Yes	627 (68.8%)	394 (71.0%)	233 (65.4%)
Years as seafarer, n (%)			
< 11 years	388 (43.1%)	243 (44.4%)	145 (41.1%)
≥ 11 years	512 (56.9%)	304 (55.6%)	208 (58.9%)
Months on vessel, n (%)			
< 5 months	448 (50.5%)	166 (31.0%)	282 (80.3%)
≥ 5 months	439 (49.5%)	370 (69.0%)	69 (19.7%)

Due to incomplete responses in the questionnaires, the total sum of absolute frequencies may not always precisely match the overall frequency of the distinguishing categories. However, the percentage figures are consistently reported based on 100% of the respondents for each respective aspect

Marital status was grouped into “Single”, “Married/Partnership”, and “Divorced/Separated” categories to simplify the analysis while capturing relational support systems, which may influence resilience. The majority of seafarers were in a committed relationship or married (75.5%), with a similar distribution between officers and ratings (77.6% and 74.2%). 627 seafarers reported having children (68.8%). The percentage of seafarers who had been working as seafarers for at least eleven years was similar in the groups of officers and ratings (58.9% and 55.6%). The months on board at the time of the survey could be entered as free text. The average was 5.3 months, which is why the binary grouping was done for less than five months and at least five months on board, to reflect typical voyage durations and contract structures in maritime employment. Notably, officers, on average, spent significantly less time on board than ratings; in the ratings’ group, 69.0% had been on board for at least five months at the time of the survey, while in the officers’ group, this percentage was 19.7% (*p* < 0.001).

### Questionnaires

The analyzed questions are part of a comprehensive questionnaire divided into sections, comprising a total of 33 questions, including questions about resilience, stress perception, depression, hearing music (as one highly important coping strategy among seafarers) [10] and demographics. The questionnaire included both dichotomous and categorical response formats. Demographic data were collected using eight questions (rank, age, sex, family status, children, months on board, years as seafarer and nationality).

Information on the resilience of seafarers, stress levels, and depression was obtained through established standardized questionnaires as seen below. Participation in the study was voluntary, and participants were informed at the beginning of the survey through an information sheet that the survey was anonymous and voluntary, obtaining their permission. A total of 21 days was given for participants to complete the questionnaire.

### Resilience

The resilience of the seafarer was assessed using the Brief Resilience Coping Scale (BRCS), which measures the ability to cope with problems in stressful situations. The questionnaire consists of four items, for example “I actively look for ways to replace the losses I encounter in life” and does not include a timeframe. Responses are given on a 5-point Likert scale ranging from 1 (does not describe me at all) to 5 (describes me very well), where a higher score implies higher resilience (ranging from four to 20). In this study, individuals scoring ≤ 13 on the BRCS were classified as low resilient copers, those scoring > 13 and ≤ 16 were assigned to the medium resilient copers, and those scoring > 16 were classified as high resilient copers [11]. For reasons of clarity, the group of medium and high resilient copers were partly combined in this study.

The Cronbach’s alpha of this test was reported in the study by Kocalevent et al. (2013) with α = 0.78 [12]. In the latter study a mean value of 14.9 (SD 3.2) for the male group was observed.

### Stress

The stress perception of the seafarers was measured using the Perceived Stress Scale (PSS), by asking for feelings and thoughts during the last month. The scale consists of ten questions, rated on a 5-point Likert scale ranging from 0 (never) to 4 (very often), where a higher score indicates a higher level of perceived stress (ranging from 0 to 40). In this study individuals with a PSS-10 score below 14 were assigned to the low sense of stress group, persons with a PSS-10 score ≥ 14 and < 27 were classified into the moderate sense of stress group and persons scoring ≥ 27 on the PSS were assigned to the high sense of stress group [13]. In the study by Cohen et al. (2012) Cronbach’s alpha of 0.78 and 0.91 were reported for the PSS-10 [14]. In this study, the seafarers with medium and high stress were partly combined.

### Depression

The tendency of the seafarers towards depression was determined using the Patient Health Questionnaire (PHQ-9). The scale consists of nine questions, rated on a 4-point Likert scale ranging from 0 (not at all) to 3 (nearly every day). A higher score implies a higher tendency towards depression, where 0 is the lowest possible value and 27 is the highest. In this study individuals with an PHQ-9 score below five were considered not depressed [15]. The Cronbach’s alpha of the PHQ-9 was reported with 0.89 in the study by Kroenke et al. [16]. 

### Statistics

The statistical analysis of the questionnaire data was performed using IBM SPSS Statistics version 27.0.1.0.

Pearson Chi-squared test was applied to assess the statistical significance among the data groups. The Odds Ratio (OR) was calculated with 95% confidence intervals using binary logistic regression. An adjustment was performed by age and ethnicity. The statistical tests were conducted with a significance level (α) of 0.05. Spearman correlation was used to identify the relationship between resilience, stress and depression.

## Results

### General level of resilience, stress and depression among seafarers and influencing factors

In the overall sample of seafarers, the average BRCS score was 15.3 (SD 3.1), on a scale ranging from 4 to 20 points. Approximately 26.6% (240 seafarers) were categorized as low resilient copers (BRCS score ≤ 13), while 44.9% (405 seafarers) were classified as medium resilient copers (score > 13 and ≤ 16), and 28.5% (257 seafarers) were considered high resilient copers (score > 16). Regarding the PSS-10 scores, the average among all investigated seafarers was 14.1 (SD 5.3) out of a possible 40 points. Within this sample, 40.1% (362 seafarers) were placed in the low stress perception group, 59.8% (540 seafarers) in the moderate stress perception group, and only 0.1% (one seafarer) in the high stress perception group. For clarity and consistency, binary evaluation was employed for both resilience scores and stress perceptions in the tables and subsequent analyses. It must be noted, however, that in the trivariate analysis of resilience, additional significant findings emerge concerning years as a seafarer, months on board, and presence of children. These influencing factors are summarized in Table S1 (see Additional File 1).

In terms of the PHQ-9 scores, the average among seafarers was 2.0 (SD 2.9) out of a possible 27 points. The majority, 85.5% (766 seafarers), were classified as not depressed, while 14.5% (130 seafarers) were categorized as depressed.

The origin as an influencing factor on resilience showed a significant association; European seafarers more often expressed a medium/high resilience than seafarers from non-Europe (78.6% vs. 70.3%; *p* = 0.023). Correspondingly, multivariate analysis demonstrated that European crew members were more likely belonging to the “medium/high resilient copers” than the non-Europeans (aOR 1.52; 95% CI (1.04–2.24)).

Crew members with easy access to music via the internet on board significantly more frequently displayed medium to high resilience (75.7% vs. 39.3%; *p* < 0.001). Multivariate analysis also demonstrated that belonging to the “medium/high resilient copers” group was strongly associated with having an easy access to music on board (aOR 4.98; 95% CI (2.56–9.70)). In binary distinction, the age, the family status and presence of children, as well as years as seafarer and months on board did not prove to be significant influencing factors on resilience (Additional File 1).

The study also explored various factors influencing stress perception among seafarers through Chi-squared tests. Significant associations emerged, notably regarding origin since non-European seafarers reported more often medium to high stress perception than Europeans (62.0% vs. 51.5%; *p* = 0.009). Additionally, the duration spent on the vessel significantly influenced stress perception (*p* < 0.001), with those spending at least five months more often reported medium/ high stress (66.3% vs. 54.0%). Moreover, easy access to music on board significantly influenced stress perception (*p* < 0.001). Seafarers with easier access to music reported high stress levels less frequently (58.2%) compared to those with more difficult access (85.7%).

Regarding age, family status, children and years as a seafarer no significant associations could be observed (Additional File 1).

In addition, the study investigated the prevalence of depression among seafarers and its association with various demographic and occupational factors. Analysis by age revealed a significant difference in depression prevalence between younger and older seafarers (*p* = 0.004). Seafarers under 40 years old exhibited a higher prevalence of depression compared to those 40 years or older (17.6% vs. 10.7%). Furthermore, the duration spent on the current vessel was significantly associated with depression prevalence (*p* < 0.001). Seafarers spending five or more months on board had a higher prevalence of depression compared to those spending less time (19.6% vs. 9.7%). The origin, family status, presence of children, years as seafarer and access to music did not reveal a significant influence on depression frequencies among seafarers (Additional File 1).

### Differences between officers and ratings in resilience, stress and depression

Among the 547 ratings giving information about their resilience, significantly more were classified as “low resilient” compared to the 355 officers (30.7% vs. 20.3%; *p* < 0.001). Consequently, medium to high resilience was observed significantly more often among officers (79.7% vs. 69.3%, *p* < 0.001) (Table 2). As 65.1% of officers were Europeans and 95.6% of ratings came from countries outside Europe an obvious association between occupational and cultural groups exists. Therefore, a logistic regression was conducted revealing significantly more frequently medium/higher resilience among officers (OR 1.74; 95% CI (1.27–2.39)). After adjusting for age and ethnicity the association between occupation group and resilience remained significant (aOR 1.63; 95% CI (1.02–2.61)).

Among the 550 ratings giving information about their stress-perception, significantly less were classified having a “low stress-perception” compared to the 353 officers (35.6% vs. 46.7%; *p* = 0.001) (Table 2). Consequently, a “moderate to high stress-perception” was significantly more often seen in ratings.

Considering the results of the PHQ-9, there is no difference in the findings between officers and ratings.

**Table 2:** Differences between occupational groups regarding resilience, stress and depression

	Officers	Ratings	p*
Resilience (BRCS)**			**< 0.001**
Low resilient copers	72 (20.3%)	168 (30.7%)	
Medium/high resilient copers	283 (79.7%)	379 (69.3%)	
Stress (PSS-10)**			**< 0.001**
Low stress perception	165 (46.7%)	197 (35.8%)	
Moderate/high stress perception	188 (53.3%)	353 (64.2%)	
Depression (PHQ-9)			0.608
No depression	301 (86.2%)	465 (85.0%)	
Depression	48 (13.8%)	82 (15.0%)	

p* = Chi-squared test, ** = significant groups

### Association between resilience, stress and depression

Crew members with a tendency towards depression (according to PHQ-9) were significantly more often classified as “low-resilient” (36.3% vs. 25.7%; *p* = 0.006).

The logistic regression indicated that depression is significantly less common in seafarers with higher resilience (OR 0.58; 95% CI (0.39–0.86)). After adjusting for stress perception (PSS-10) the association between resilience and tendency towards depression did not remain significant (aOR 0.74; 95% CI (0.49–1.11)) (Table 3).

Crew members with a moderate to high stress perception significantly more often showed a tendency towards depression (22.2% vs. 3.6%; *p* < 0.001). A tendency towards depression was also significantly more often observed among seafarers with moderate to high stress perception in logistic regression (OR 7.53; 95% CI (4.17–13.60)). After adjusting for resilience the association between stress perception and a tendency towards depression remained significant (aOR 7.30; 95% CI (4.03–13.21)).

Seafarers with a low perception of stress were significantly more often associated with high resilience than seafarers with a moderate to high perception of stress (39.3% vs. 21.7%; *p* < 0.001). In the logistic regression a high resilience was significantly less often seen in seafarers with a moderate to high stress perception (OR 0.47; 95% CI (0.34–0.64)). After adjusting for tendency towards depression the association remained significant (aOR 0.48; 95% CI (0.34–0.67)) (Table 3).

BRCS-Score and PSS-Score correlated moderately (Spearmans ρ=-0.305, *p* < 0.001). Similarly, the correlation between PSS-10 score and PHQ-9 score was significant (Spearmans ρ = 0.333, *p* < 0.001). BRCS-Score and PHQ-9-Score showed a weaker correlation (Spearmans ρ=-0.199, *p* < 0.001).

**Table 3:** Odds Ratio for resilience, stress and depression

	Depression (PHQ-9)		Stress (PSS-10)	
	OR	aOR	OR	aOR
Resilience (BRCS)	0.58 (0.39–0.86)	0.74 (0.49–1.11)*	0.47 (0.34–0.64)	0.48 (0.34–0.67)***
Stress (PSS-10).. depression.	7.53 (4.17–13.60)	7.30 (4.03–13.21)**		

*adjusted for stress perception, **adjusted for resilience, ***adjusted for tendency towards

## Discussion

Seafaring is regarded as a high stress occupation that may affect the mental health of seafarers and can lead to depression [17, 18]. Thus, it is of high importance to increase the resources of shipping crews and improve their resilience [19]. The aim of the present study is to identify and illustrate the relationships between resilience, stress levels, and depression among seafarers. Additionally, the study sought to determine influencing factors and analyze differences related to occupational groups.

### General level of resilience, stress and depression among seafarers and influencing factors

The present findings suggest that according to BRCS the majority of seafarers (73.4%) demonstrate medium to high levels of resilient coping, indicating a capacity to deal with stressors and adversity at sea. However, it is important to note that the BRCS assesses resilient coping strategies rather than the broader, multidimensional process of resilience, which encompasses long-term adaptation and dynamic psychological processes. Therefore, these results reflect coping strategies rather than a full representation of resilience in this population. Since the BRCS has not been demonstrably used in seafaring during the last 20 years, there is no comparative collective from shipping to compare these data [9]. Therefore, a comparison collective from the general population ashore can only be used. In the study by Kocalevent et al. (2017) a nationwide survey in Germany was conducted [12]. The mean BRCS score among men was 14.9 (SD 3.2) [12], indicating a similar value than that collected in this study from seafarers (15.3). This suggests a similar resilience in seafarers compared to the general population.

Furthermore, a considerable portion of seafarers (59.9%) experience medium to high levels of stress, with a notable percentage (14.5%) exhibiting symptoms of depression. In the study by Lefkowitz et al. (2019), a depression prevalence of 25% was found among seafarers, which is notably higher than the measured value in this study [20]. In a study by Baygi et al. (2022), the frequency of depressive symptoms among seafarers was 14.1%, which corresponds to the same manifestation of depression as in this study (14.0%) [21]. However, compared to a reference group from Germany, where the prevalence of depression in the general population is 6%, the value measured in this study is still higher than in the general population [22]. 

According to present findings, several factors emerge as potential influencers of resilience among seafarers. The association between current stay on board (months on vessel) and resilience suggests that the present stressful working situation has a relevant impact on resilience of the seafaring personnel. Correspondingly, it has been shown that seafarers with longer duration of employment on board more often develop depression or mental health problems [17]. Seafarers who have been in the maritime profession for a long time also frequently report lower resilient coping in this study. This could be attributed to a high accumulation of stress and ongoing exhaustion [23]. 

Furthermore, seafarers with children often exhibited lower resilience with a tendency towards significancy, which could be attributed, possibly, to increased homesickness, as they may develop longing for their family more quickly. It should also be noted that in the trivariate analysis of resilience, which has not been considered here for the sake of clarity, months on board, time as a seafarer, and having children were identified as significant influencing factors.

The opportunities on board the ship to enjoy ones leisure time are often limited. Aside from board games, sports such as table tennis and the gym, or watching movies, karaoke is also a very popular option, especially among Filipinos, to make use of their free time. According to the authors` own experience, many seafarers, especially non-European crew members, practice karaoke in their free time at sea to relax and use music as an effective tool to expand their personal resources [24]. Music provides a diverse way to relax and fill leisure time, as it can be enjoyed together, for example, during karaoke, or alone, through devices like a mobile phone when colleagues are not available to spend leisure time with [10, 25]. Thus, access to music via internet is highly important on board. The present study highlights that an easy access to music constitute a crucial source for relaxation and is strongly associated with higher resilience, suggesting the importance of recreational activities in bolstering resilience at sea. In a study by Fink et al. (2021) music also emerged as an important resource in coping with emotional and social stressors [26]. In this context, the cultural background of the seafarers also emerged as a significant influencing factor on resilience, with seafarers of European background significantly more often reporting medium/high resilient coping compared to seafarers of non-European background. Thus, it is important to understand that the relaxation preferences and the resilience among the multicultural crews on board is very heterogeneous and need to be considered when planning health promotions programs on vessels.

In terms of stress perception, non-European seafarers were significantly more likely to report moderate to high stress perception compared to European seafarers (62.0% vs. 51.5%). Moreover, spending more time on the vessel was notably associated with higher stress perception. The association between stress perception and time spent on the vessel per month suggests that prolonged exposure to the maritime environment could exacerbate stress levels [8, 17]. This could be due to the inherent challenges of life at sea, including isolation, demanding work schedules, and the unpredictable nature of shipping.

Furthermore, easier access to music was also associated with lower levels of stress among seafarers, indicating the availability of music as a coping mechanism as it provides a source of relaxation and distraction from the rigors of their work. However, age, years as seafarers, presence of children and family status showed no significant association with stress perception.

Regarding depression prevalence, age was the only demographic factor significantly associated with depression prevalence. Younger seafarer more often reported signs of depression than seafarers aged 40 or older (17.6% vs. 10.7%, *p* = 0.004). In the study by Lee et al. (2023), it was shown that the prevalence of depression in the USA was highest among the age group of 18-24-year-olds, at 21.5% [27]. This suggests a generally higher prevalence of depression among younger people as confirmed in the present study. A healthy-worker effect can be discussed as a possible explanation for this finding [28]. The older and therefore more life-experienced workers typically have a longer professional career and have become accustomed to their (potentially stressful) work environment, while the younger workers may leave their jobs in the near future when feeling exhausted and overworked. However, in the present study, surprisingly, no connection was found between the length of professional experience (representing the chronic work-related stress) and the seafarers’ tendency to depression.

However, spending five or more months on the vessel indicating the seafarers` current stress level was significantly associated with a higher prevalence of depression, implying that prolonged periods away from home and family may increase feelings of loneliness and isolation, contributing to depressive symptoms [29]. Interestingly, rank and ease of access to music on board did not exhibit significant associations with depression prevalence. Origin, family status, and years of experience as a seafarer did not show significant associations, suggesting that depression among seafarers may be influenced by a combination of occupational and environmental factors rather than solely demographic ones.

### Differences between officers and ratings in resilience, stress, and depression

In this study, officers tend to demonstrate higher resilience more often compared to ratings. The findings also reveal that ratings more often perceive subjectively higher stress levels compared to officers. This difference in stress perception between officers and ratings may be due to the higher resilience of officers or stem from variations in job roles, responsibilities, and work environments onboard ships. Officers typically have more administrative and strategic tasks, which are demanding but more structured and predictable, also contributing to higher resilience. They also have shorter deployment times, perform less physically demanding tasks, generally work in better conditions and have greater access to social support networks, which help reduce stress. Ratings, on the other hand, often perform physically demanding and less predictable tasks in more challenging environments, like engine rooms or decks, leading to higher stress levels. They may also have less communication options to stay in touch with home and show a higher risk of burnout [30], which could also be explained by their higher stress perception.

However, no significant difference is observed in depression rates between officers and ratings. This suggests that the overall strain of life at sea might generally have a strong impact on mental health, regardless of job role.

### Association between resilience, stress, and depression

**Fig. 1:**
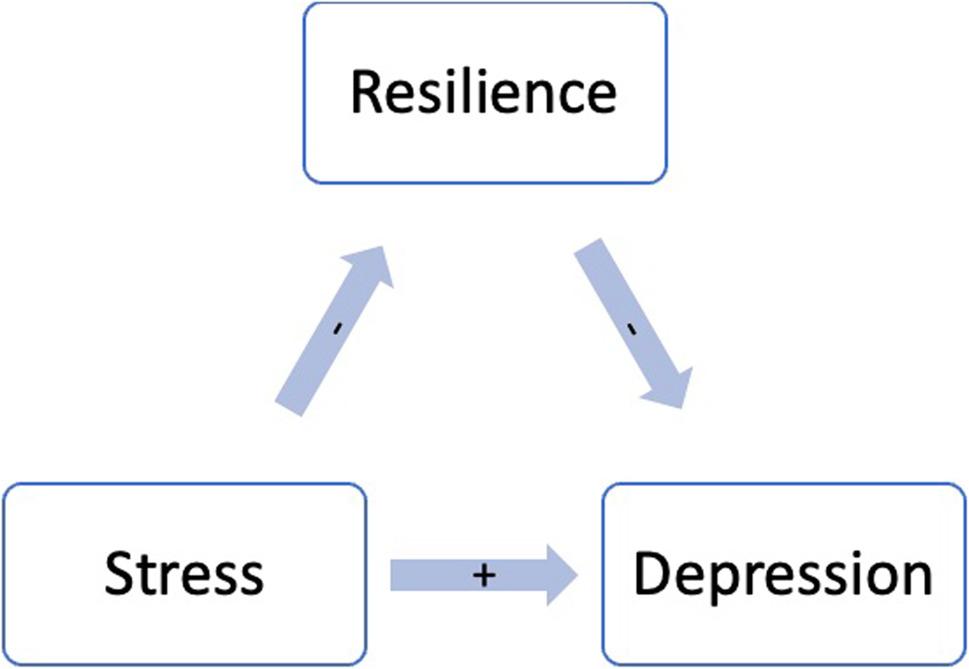
Conceptual mediation model illustrating the indirect effect of stress on depression via resilience

The present study reveals a significant mediation pathway between resilience, stress perception, and depression among seafarers. Notably, individuals with higher resilience are less likely to experience depression, highlighting a protective role of resilience against mental health issues (Fig. 1). Higher stress perception is strongly associated with an increased tendency towards depression, underscoring the importance of addressing stressors in promoting mental well-being among seafarers.

The findings in this study align with existing literature suggesting that prolonged stress events contribute to the onset of depression. Stress can lead to an increase in hormone production such as cortisol and a decrease in the availability of neurotransmitters like serotonin and dopamine in the brain, which are associated with depressive symptoms [6]. Resilience plays a crucial role in coping with stress and stress-related disorders. Individuals with higher resilience exhibit better abilities to manage stressors, thereby reducing the likelihood of stress-induced depression [7]. 

Longitudinal studies have demonstrated that resilience serves a protective function concerning mood states. Higher levels of resilience are associated with better capabilities to cope with and counteract depressive symptoms [31, 32]. Moreover, individuals with lower resilience tend to perceive higher levels of stress. Lower resilience often correlates with increased susceptibility to stress and subsequently leads to a rise in depressive symptoms [7]. 

Our findings support a mediation model in which resilience plays a central role in the pathway from stress to depression. Specifically, resilience may serve as a protective psychological resource that attenuates the negative impact of stress on mental health (Fig. 1). This aligns with prior research emphasizing the importance of resilience as a mechanism that helps individuals maintain psychological well-being under stress [7]. Stress and depression have a significant impact on each other, as do stress and resilience. These relationships suggest that resilience and depression are not directly correlated but are connected through their influence on stress perception. Thus, decreased resilience can lead to higher perceived stress levels, which can eventually contribute to the development of depression. Therefore, reduced resilience, through its negative impact on stress perception, can influence the development of depression. Conversely, depression could also lead to higher perceived stress levels in daily life, resulting in an overall decrease in resilience. While these associations are statistically significant, the cross-sectional nature of the data limits interpretation regarding directionality or changes over time. Recent research further reinforces this relationship. A cross-sectional study by Safiye et al. demonstrated that higher levels of resilience and mentalizing ability were associated with better mental health outcomes among healthcare workers during the COVID-19 pandemic. The study identified a significant inverse relationship between resilience and both depression and perceived stress, suggesting that enhancing resilience may serve as a key protective factor in challenging environments. Although the study focused on healthcare workers, the findings are highly transferable to the maritime sector, where personnel similarly operate under prolonged stress and isolation [33]. 

### Implications for intervention and future research

The present findings underscore the need for targeted interventions aimed at enhancing resilience and mitigating stressors among seafarers as primary prevention. Interventions could include promoting psychosocial support programs and implementing stress management strategies on board. Additionally, the PSS-10 could be used as a screening tool on board, as this questionnaire can identify seafarers who experience high levels of stress, allowing them to be targeted for support. Shipping crews with high stress levels significantly more often showed lower resilience and a tendency toward depression [34–37]. With targeted intervention, resilience among seafarers could be increased, stress levels reduced, and thereby protection against the development of depression could be provided. The Brief Resilience Coping Scale (BRCS) could be a valuable tool for assessing and monitoring resilience among seafarers. Its brevity makes it convenient for use in busy maritime settings, and its standardized nature allows for easy comparison of resilience levels across different groups and over time. By incorporating the BRCS into routine health assessments or mental health screenings, shipping companies can gain insights into the resilience levels of their crews and identify individuals who may benefit from additional support or intervention. Additionally, tracking changes in resilience scores over time can help evaluate the effectiveness of resilience-building initiatives. Additionally, the PHQ-9 could offer a means of secondary prevention through regular routine screening testing, by identifying crew members who already show symptoms of depression. This would allow for targeted individual support to protect their mental health and prevent them from developing a severe, full-blown depression. Recognizing the cultural diversity among seafaring populations is essential when designing interventions and support programs. Cultural preferences and practices, such as leisure activities, may influence resilience and coping mechanisms and should be considered in intervention planning. Furthermore, future research should delve deeper into the interplay between individual, organizational, and environmental factors in shaping resilience among seafarers, with a focus on developing evidence-based interventions to support their mental well-being. Longitudinal studies are needed to assess the long-term effects of interventions and changes in mental health outcomes over time. This would provide valuable insights into the effectiveness of interventions and help refine strategies for promoting mental well-being among seafarers. Future research should also aim to quantify the specific effect size of modifiable factors like music access through multivariate analyses, to better understand their relative importance in mental health promotion strategies for seafarers.

### Strengths and limitations of the study

The study exhibits several strengths and limitations. On the positive site it is the first study worldwide examining the resilience of seafarers in respect of different occupational groups. It explores key demographic and professional factors that impact mental health parameters. Moreover, the use of standardized instruments such as the Brief Resilience Coping Scale (BRCS), Perceived Stress Scale (PSS-10), and Patient Health Questionnaire (PHQ-9) lends credibility and reliability to the measurements. Additionally, the study’s findings have practical implications for designing targeted interventions to enhance the well-being of seafarers. However, the study also has limitations. Being a cross-sectional study, it cannot establish causality, and its findings may not be generalizable beyond the specific shipping company surveyed. Given the evolving nature of resilience, the cross-sectional design may not capture its development over time. Longitudinal studies are necessary to fully understand the temporal dynamics between resilience, stress, and depression. The reliance on self-report measures introduces potential biases, and the lack of longitudinal data prevents the assessment of long-term effects of interventions. Moreover, unmeasured variables such as specific working conditions and social support onboard may influence mental health outcomes. Although standardized questionnaires were used, it must be noted that the BRCS is a resilience assessment tool that is very brief, consisting of only four items, and thus may not have captured the resilience of seafarers as comprehensively as longer questionnaires such as the Dispositional-Resilience-Scale (DRS-15). Additionally, it has not been previously utilized in maritime settings, hence there are no comparative datasets available [9]. 

## Conclusion

In conclusion, this study sheds light on the complex relationship between resilience, stress perception, and depression among seafarers. It underscores the importance of understanding the unique challenges faced by this population and the need for targeted interventions to promote their mental well-being. By identifying factors influencing resilience and stress perception, such as job role and cultural background, this study provides valuable insights for designing effective support programs. Moving forward, there is a critical need for further research to explore the underlying mechanisms driving these associations and to develop evidence-based interventions tailored to the specific needs of seafarers. By addressing these issues, stakeholders can better support the mental health of seafarers and promote a safer and more sustainable maritime industry.

## Supplementary Information

Supplementary Material 1.

## Data Availability

According to the informed consent form and ethics application, we are not allowed to publish the dataset, as given the rare circumstances (e.g., female captain), an individual (e.g., from the involved shipping company) could be identified. The datasets used and analyzed during the current study are available from the corresponding author on reasonable request.
