# Supplementary material for: Resilience of seafarers depending on occupational groups
Source: BMC Public Health. 2026 Mar 31;26:1171. doi: 10.1186/s12889-026-27133-6 (PMC13063892; doi:10.1186/s12889-026-27133-6)
Supplement: Supplementary file 1 — Supplementary Material 1. [file 12889_2026_27133_MOESM1_ESM.pdf]

Table 2 Influencing factors on resilience, stress perception and depression

	Low resilient copers	Medium/high resilient copers	p*	Low stress perception	Moderate/high stress perception	p*	No depression	Depression	p*
**Total group**	240 (26.6%)	662 (73.4%)		362 (40.1%)	541 (59.9%)		766 (85.5%)	130 (14.5%)	
**Age**, n (%)			0.110			0.535			**0.004**
< 40 years	119 (24.4%)	368 (75.6%)		199 (40.8%)	289 (59.2%)		402 (82.4%)	86 (17.6%)	
≥ 40 years	114 (29.2%)	276 (70.8%)		151 (38.7%)	239 (61.3%)		342 (89.3%)	41 (10.7%)	
**Origin**, n (%)			**0.023**			**0.009**			0.423
Non-European	154 (29.7%)	364 (70.3%)		197 (38.0%)	322 (62.0%)		437 (84.9%)	78 (15.1%)	
European	44 (21.4%)	162 (78.6%)		100 (48.5%)	106 (51.5%)		177 (87.2%)	26 (12.8%)	
**Family status**, n (%)			0.507			0.417			0.223
Single	48 (23.8%)	154 (76.2%)		88 (43.6%)	114 (56.4%)		168 (82.4%)	36 (17.6%)	
Married/Partnership	184 (27.6%)	483 (72.4%)		258 (38.8%)	407 (61.2%)		568 (86.6%)	88 (13.4%)	
Divorced/Separated	3 (21.4%)	11 (78.6%)		7 (46.7%)	8 (53.3%)		14 (93.3%)	1 (6.7%)	
**Presence of children**, n (%)			0.066			0.152			0.170
No	61 (22.0%)	216 (78.0%)		122 (43.7%)	157 (56.3%)		230 (83.0%)	47 (17.0%)	
Yes	170 (27.9%)	440 (72.1%)		235 (38.7%)	373 (61.3%)		521 (86.5%)	81 (13.5%)	
**Years as seafarer**, n (%)			0.086			0.437			0.350
< 11 years	89 (23.5%)	289 (76.5%)		156 (41.3%)	222 (58.7%)		321 (84.5%)	59 (15.5%)	
≥ 11 years	143 (28.7%)	355 (71.3%)		193 (38.7%)	306 (61.3%)		424 (86.7%)	65 (13.3%)	
**Months on vessel**, n (%)			0.073			**<0.001**			**<0.001**
< 5 months	103 (23.4%)	338 (76.6%)		202 (46.0%)	237 (54.0%)		392 (90.3%)	42 (9.7%)	
≥ 5 months	122 (28.7%)	303 (71.3%)		144 (33.7%)	283 (66.3%)		341 (80.4%)	83 (19.6%)	
**Access to music on board**, n (%)			**<0.001**			**<0.001**			0.279
Easy	206 (24.3%)	640 (75.7%)		354 (41.8%)	493 (58.2%)		718 (85.2%)	125 (14.8%)	
Not Easy	34 (60.7%)	22 (39.3%)		8 (14.3%)	48 (85.7%)		48 (90.6%)	5 (9.4%)	

p* = Chi-squared test
